# Screening at a Federally Qualified Health Center in the Midwest for Hepatitis C Among People Who Inject Drugs, 2019–2020

**DOI:** 10.5888/pcd18.200604

**Published:** 2021-07-15

**Authors:** Melissa Perkins, Amber Slevin, Mark A. Strand, Daniel Freisner

**Affiliations:** 1 North Dakota State University, Fargo, North Dakota; 2 Pharmacy Practice Department, North Dakota State University, Fargo, North Dakota; 3 Family HealthCare, Fargo, North Dakota; 4 Department of Pharmacy Practice and Department of Public Health, North Dakota State University, Fargo, North Dakota

## Abstract

**Introduction:**

Hepatitis C virus (HCV) infection is a public health epidemic. People who inject drugs (PWID) are at high risk for transmitting and contracting HCV. The objective of this study was to assess the effectiveness of a multifaceted intervention at a federally qualified health center in the US Midwest to improve HCV screening rates among PWID.

**Methods:**

A prospective quality improvement initiative was conducted to increase the proportion of PWID screened for HCV. Inclusion criteria consisted of being seen by a primary care provider from April 16, 2019, through February 28, 2020, being aged 18 years or older, and confirmation of intravenous drug use. PWID status was confirmed by reviewing electronic health records. The multifaceted intervention consisted of educational sessions for the health care team and workflow changes. We analyzed the proportion of patients screened preintervention and postintervention by using χ^2^ tests.

**Results:**

Of 742 patients who met the inclusion criteria, the proportion of PWID screened preintervention was 59.6% (n = 329) and the proportion of PWID screened postintervention was 65.1% (n = 283), increasing the screening rate by 5.5 percentage points. A χ^2^ test of homogeneity indicated a significant relationship between the preintervention and postintervention periods, and screening outcomes (*P* < .001).

**Conclusion:**

This multifaceted intervention to increase HCV screening resulted in a modest increase in the proportion of PWID screened. Consistent and health care system–wide screening approaches are needed to optimize the potential of HCV treatment and cure options now available.

**Summary**

**What is already known on this topic?**

Hepatitis C virus (HCV) is prevalent among and transmitted by people who inject drugs (PWID). The current rates of HCV screening and treatment are insufficient to affect population-wide HCV outcomes, particularly among PWID.

**What is added by this report?**

Our study analyzed a multifaceted intervention in a federally qualified health center that resulted in a modest increase in the proportion of PWID screened for HCV. We also found possible barriers to optimizing HCV care.

**What are the implications for public health practice?**

Risk-based HCV screening presents challenges. While new universal screening guidelines may overcome some barriers, additional efforts are needed to diagnose and ultimately treat HCV among PWID to reduce population-wide prevalence and transmission.

## Introduction

Hepatitis C virus (HCV) is a blood-borne pathogen spread commonly through sharing syringes. People who inject drugs (PWID) are at high risk of contracting HCV; the global seroprevalence of HCV is 52.9% among PWID (1,2). Although the World Health Organization suggests that HCV could be eliminated as a public health threat by 2030, work remains to meet this aim, particularly among PWID (2). One study estimated that only 7.7% of commercially insured PWID in the US were screened for HCV from 2010 to 2017 (3). According to Healthy People 2030, increasing the knowledge of an HCV infection from 55.6 percent to 74.2 percent will meaningfully improve population-wide HCV outcomes (4).

**Figure 1:**
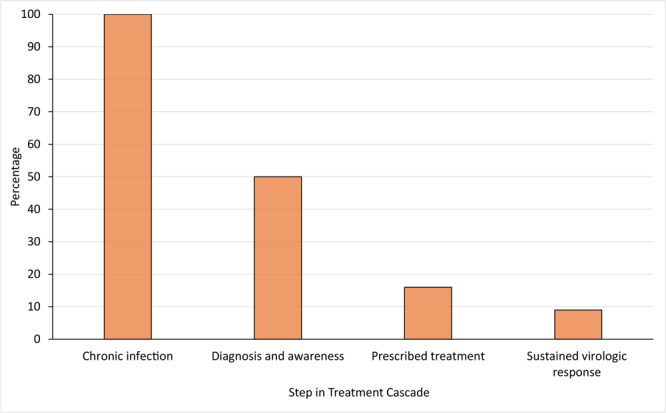
Estimated percentage of people with hepatitis C virus (HCV) at each step of the care cascade, US, 2014. Approximately 3.5 million people in the US have chronic HCV infection. Figure adapted with permission from Yehia et al (7).

Essential to the eradication of HCV is optimizing steps in the HCV care cascade, which includes diagnosis, curative therapy initiation, and confirmation of sustained virologic response (ie, cure). The number of patients sharply declines at each step of the care cascade in the US; the sharpest decline occurs between infection and diagnosis (Figure 1) (5). In 2015, 71 million people had HCV worldwide. Approximately 14 million were diagnosed with HCV (20%), and 5 million were treated (7%). In 2019, the number of HCV infections worldwide remained at 71 million (6); this number is stagnant globally despite the availability of curative therapy (7).

**Table:** 

Step in care cascade	Percentage
Chronic infection	100
Diagnosis and awareness	50
Prescribed treatment	16
Sustained virologic response	9

The optimization of the care cascade among PWID requires integration of PWID identification and documentation into standard medical care, yet best practices for such integration are elusive (8). The primary objective of this study was to determine the proportion of PWID screened for HCV at a federally qualified health center (FQHC) in the Midwest and measure the effectiveness of an intervention to increase screening among PWID. The study hypothesis was that an evidence-based, multifaceted intervention aimed at the health care team responsible for conducting HCV screening would increase the proportion of PWID screened for HCV.

## Methods

A prospective quality improvement initiative was conducted at an FQHC primary care clinic to identify and improve HCV screening among PWID. The intervention consisted of educational sessions for the health care team (nurses, physicians, advanced practice providers, and the clinic-based pharmacy staff) and clinic workflow changes. Education covered the prevalence and incidence of HCV infection, screening guidelines, and new clinic-specific tools to support screening. Workflow changes were implemented to alert medical providers of people who needed HCV testing via electronic health records (EHRs) and patient visit forms.

The North Dakota State University Institutional Review Board approved this study. We defined the primary outcome as an increase in the proportion of PWID screened for HCV after implementation of a multifaceted intervention in October 2019. We aimed the intervention at the FQHC health care team rather than patients. Not all patients were seen in both preintervention and postintervention periods; the opportunity to complete screening occurred when patients presented for medical care. The study focused on the success of the intervention and the increase in the percentage of patients screened, whether seen preintervention or not.

Inclusion criteria for participation were 1) being seen by a primary care provider from April 16, 2019, through February 28, 2020, 2) being aged 18 years or older, and 3) confirmation of current or former intravenous drug use (IVDU). Preintervention data collection took place from April 16, 2019, through October 29, 2019; postintervention data collection took place October 30, 2019, through February 28, 2020. We confirmed PWID status by reviewing the EHR for 2 criteria: 1) an *International Classification of Diseases, 10th Revision, Clinical Modification* (ICD-10-CM) code for illicit drug use or 2) the nursing form completed when a patient attended a medical visit indicated that the patient reported current or former IVDU. We then systematically verified IVDU history for each patient by examining the EHR for the presence of free text notes in either the patients’ problem list or patient encounter documentation indicating a medical provider had confirmed current or former IVDU or the nursing form documented IVDU. The nursing form contained the question, “Does the patient currently or have they ever used IV drugs?” When IVDU was not documented during the medical visit, we reviewed patient medical records from outside facilities available within the patient’s EHR for any indication of IVDU.

### Data collection

In October 2018 the clinic implemented a new EHR-based questionnaire for nurses to increase identification and documentation of IVDU at each clinic visit, given the lack of ICD-10-CM code specific to injection of illicit drugs. This initiative to improve the identification and documentation of PWID was implemented before the study period to ensure PWID were properly identified before assessing screening proportions. The questionnaire prompted the nurse to ask what kind of drug the patient used and the route of administration. Data collected from October 2018 to April 2019 showed little improvement in PWID documentation; many EHRs indicated illicit drug use but did not identify a route of administration. An explicit question was then added in April 2019 to the EHR-based questionnaire; nurses were required to ask patients if they had ever used intravenous drugs. Anecdotally, after this question was added, PWID identification and information route of administration increased.

We initiated preintervention data collection on April 16, 2019, to establish a baseline proportion of PWID screened for HCV. We examined EHRs for completion of HCV screening on at least 1 occasion. We considered screening complete if an HCV antibody or HCV RNA quantitative polymerase chain reaction test had been completed. If we found no results of a test received in the clinic, we searched the EHR for records of screening conducted outside the clinic. We used the same process to collect data during postintervention.

### Intervention

During the last week of October 2019, a multifaceted intervention was conducted to improve screening, expand medical provider recommendation, and increase HCV screening tests ordered. The intervention consisted of an educational component as well as clinic-specific workflow changes to support HCV screening. An infectious disease physician, a pharmacist specializing in viral hepatitis, and a Doctor of Nursing practice student conducted 2 on-site educational presentations for the health care team members. The presentations focused on the prevalence of HCV, risk-based HCV screening guidelines in place at the time, new clinical tools to support HCV screening, components of a hepatitis C toolkit created by the Centers for Disease Control and Prevention (CDC), and distribution of educational materials. Thirty-five staff members attended the 2 presentations. Changes made to the clinical workflow involved items for nurses, physicians, advanced practice providers and the clinic-based pharmacy team. We updated the preventive care visit form completed by nursing staff to identify PWID and document receipt of HCV screening according to the risk-based screening guidelines in effect at that time. The nurses completed the paper-based, preventive care form before all patient visits during postintervention. Pharmacy students prospectively reviewed EHRs and placed EHR-based alerts to medical providers in the EHRs of patients for whom screening was indicated and had not yet been completed. Medical providers were responsible for discussing HCV screening with patients and ordering tests. Because the intervention was intended to increase screening recommended at medical visits, some patients were seen preintervention only, some were seen postintervention only, and some patients were seen in both periods.

### Analysis

We adopted a general null hypothesis of no relationship between the study period (preintervention vs postintervention) and PWID screening outcomes (ie, the intervention is not effective at identifying and documenting PWID). We also adopted a null hypothesis of no relationship between patients’ demographic characteristics and either the study period or PWID screening outcomes.

We used χ^2^ tests of homogeneity to evaluate the general null hypothesis. We created cross-tabulations that disaggregated screening outcomes and patient demographic variables (sex [male or female], age, and Hispanic ethnicity [yes or no]) across preintervention and postintervention periods. Data were based on office visits scheduled by the patients, meaning that patients could have been seen in both periods or only 1 period. We used Microsoft Excel and the SPSS Statistics Package version 27 (IBM Corporation) for all statistical analyses; *P *< .05 was considered significant.

## Results

**Figure 2:**
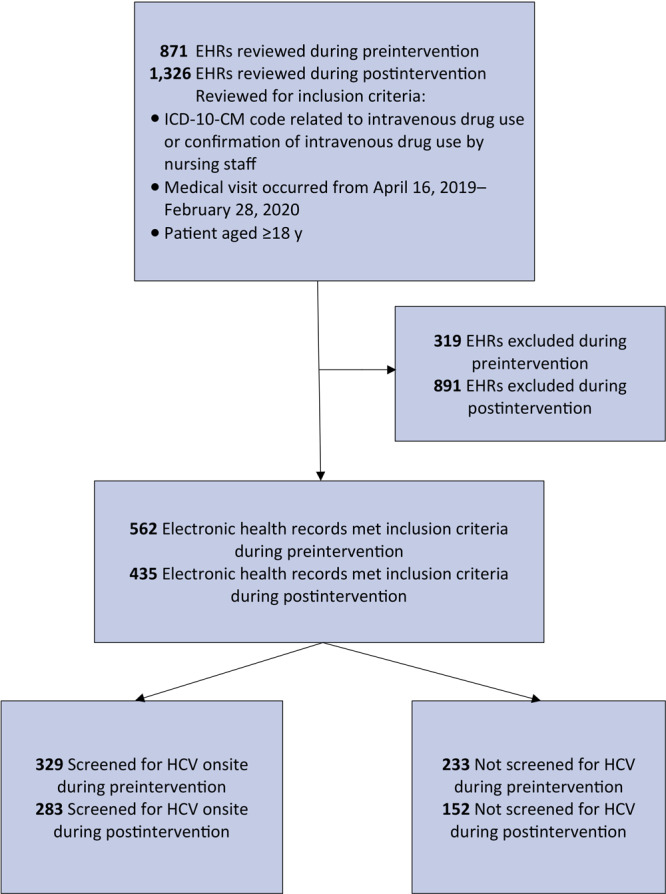
Identification of people who inject drugs and receipt of screening for hepatitis C virus at a primary care clinic in a federally qualified health center in the US Midwest. Preintervention data collection took place from April 16, 2019, through October 29, 2019; postintervention data collection took place October 30, 2019, through February 28, 2020. Abbreviations: EHR, electronic health record; HCV, hepatitis C virus; ICD-10-CM, *International Classification of Diseases, Tenth Revision, Clinical Modification*.

We identified 742 patients as PWID during the study period; 562 patients met the inclusion criteria during preintervention, and 435 patients met criteria during postintervention (Figure 2). Of the patients seen during preintervention, 245 were also seen during postintervention.

Of the 742 patients, 400 (53.9%) were men and 46 (6.2%) were Hispanic; 258 (34.8%) were aged 30 to 39 (Table 1). We found no significant differences across study periods in patient sex (*P* = .62) or ethnicity (*P* = .79). However, we found significant differences across study periods in patient age. More people in their 50s and 60s than in younger age groups were seen both preintervention and postintervention. Younger patients were more likely to be seen preintervention than postintervention or both periods. We found no significant relationship among 742 patients between HCV screening status (ie, screened or not screened) and patient sex, age, or ethnicity (Table 2).

**Table 1:** Characteristics of People Who Inject Drugs Seen at a Federally Qualified Health Center During an Intervention to Improve Screening for Hepatitis C Virus, Disaggregated by Demographic Variables, Midwestern US, 2019–2020

Characteristic	Patient Seen Preintervention Period Only (April 16, 2019–October 29, 2019)	Patient Seen Postintervention Period Only (October 30, 2019–February 28, 2020)	Patient Seen in Both Periods	Total	χ^2^ *P* Value
**Sex**					
Female	135	90	117	342	.62
Male	172	100	128	400	
Both sexes	307	190	245	742	
**Age, y**					
<30	70	56	36	162	.001
30–39	107	69	82	258	
40–49	70	38	52	160	
50–59	42	21	55	118	
≥60	18	6	20	44	
All ages	307	190	245	742	
**Ethnicity**					
Hispanic	17	12	17	46	.79
Not Hispanic	290	178	228	696	
Both ethnicities	307	190	245	742	

**Table 2:** Cumulative Hepatitis C Virus Screening Outcomes Among People Who Inject Drugs Seen at a Federally Qualified Health Center During an Intervention to Improve Screening for Hepatitis C Virus, Disaggregated by Demographic Variables, Midwestern US, 2019–2020

Characteristic	Not Screened	Screened	Total	χ^2^ *P* Value
**Sex**				
Female	138	204	342	.71
Male	156	244	400	
Both sexes	294	448	742	
**Age, y**				
<30	65	97	162	.25
30–39	105	153	258	
40–49	68	92	160	
50–59	36	82	118	
≥60	20	24	44	
All ages	294	448	742	
**Ethnicity**				
Hispanic	22	24	46	.24
Not Hispanic	272	424	696	
Both ethnicities	294	448	742	

When we disaggregated the number of patients seen and the number of patients screened by study phase (preintervention period, postintervention period, or both), we found a significant relationship between the preintervention and postintervention periods and screening outcomes. Of the 552 patients seen during preintervention, 329 (59.6%) were screened for HCV; of the 435 patients seen during postintervention, 283 (65.1%) were screened, an increase of 5.5 percentage points (Table 3). Of 190 patients not seen preintervention but seen postintervention, 103 (54.2%) were screened for HCV. Of 65 patients seen but not screened preintervention, 16 patients were screened postintervention, indicating potential barriers specific to these 65 patients. We found a high level of consistency in outcomes across patients of differing sex, age, and ethnicity (Table 4). The significance demonstrated by the χ^2^ tests appear to be driven primarily by the relationship between patient visits and screening, rather by differences in patient demographics.

**Table 3:** Number of Visits Versus Screening Outcome Among People Who Inject Drugs Seen at a Federally Qualified Health Center During an Intervention to Improve Screening for Hepatitis C Virus, Disaggregated by Demographic Variables, Midwestern US, 2019–2020

Preintervention screening outcome (n = 552)	Screening Outcome (n = 435)				
	Not Screened (n = 152)	Screened (n = 283)	Not Seen (n = 307)	Total	χ^2^ *P* Value
Not screened	65	16	142	223	<.001
Screened	0	164	165	329	
Not seen	87	103	0	190	

**Table 4:** Screening Outcomes, by Number of Visits, Sex, Age, and Ethnicity, Among People Who Inject Drugs Seen at a Federally Qualified Health Center During an Intervention to Improve Screening for Hepatitis C Virus, Disaggregated by Demographic Variables, Midwestern US, 2019–2020

Outcome	Screening Outcome (n = 435)			
	Not Screened	Screened	Total	χ^2^ *P* Value
**By no. of visits**				
Seen preintervention only	142	165	307	<.001
Seen postintervention only	87	103	190	
Seen preintervention and postintervention	65	180	245	
Total	294	448	742	
**By sex**				
Seen preintervention only				<.001
Female	65	70	135	
Male	77	95	172	
Seen postintervention only				
Female	44	46	90	
Male	43	57	100	
Seen preintervention and postintervention				
Female	29	88	117	
Male	36	92	128	
Total	294	448	742	
**By age, y**				
Seen preintervention only				.001
<30	32	38	70	
30–39	51	56	107	
40–49	31	39	70	
50–59	16	26	42	
≥60	12	6	18	
Seen postintervention only				
<30	27	29	56	
30–39	31	38	69	
40–49	20	18	38	
50–59	8	13	21	
≥60	1	5	6	
Seen preintervention and postintervention				
<30	6	30	36	
30–39	23	59	82	
40–49	17	35	52	
50–59	12	43	55	
≥60	7	13	20	
Total	294	448	742	
**By ethnicity**				
Seen preintervention only				<.001
Hispanic	8	9	17	
Not Hispanic	134	156	290	
Seen postintervention only				
Hispanic	6	6	12	
Not Hispanic	81	97	178	
Seen preintervention and postintervention				
Hispanic	8	9	17	
Not Hispanic	57	171	228	
Total	294	448	742	

## Discussion

This multifaceted intervention increased the proportion of PWID screened for HCV by 5.5 percentage points. Although this increase is modest, the proportion of PWID screened before the intervention was substantially higher than the proportion found in other studies. According to the US Preventive Services Task Force (USPSTF), the screening rate for HCV was 8.3% nationwide in community health centers during the risk-based screening era (9). A retrospective cohort study was conducted from 2012 to 2017 to estimate the screening rate of HCV among PWID at a single FHQC (10); 14% of patients diagnosed with opioid use disorder were screened for HCV. 

We found that only 7.1% of patients not screened preintervention were screened when they had a medical provider visit postintervention, indicating persistent barriers to screening. We do not know if these patients declined screening or if the provider did not recommend screening, but this finding warrants further study. One may expect that follow-up medical visits allow more time for preventive care and HCV screening would be conducted as appropriate, but we did not observe this trend. According to a meta-analysis published in 2018, a lack of patient knowledge in areas such as HCV risk factors, HCV transmission, and outcomes of untreated HCV infection was associated with being less likely to get tested (11). Self-perception of low risk for HCV by patients was associated with more cases of undiagnosed infection. Another patient barrier is fear and stigma associated with a positive HCV test result (11). Comorbid psychiatric disease and social determinants of health, including but not limited to homelessness, inadequate transportation, distrust of medical professionals, and less than a high school education, have been found to be prevalent among PWID with HCV and affect the HCV care cascade (12,13). Although we can anecdotally report that many of these factors are present in our FQHC patient population, we did not collect data on these factors.

Medical provider barriers can also affect the HCV care cascade among PWID. Such barriers could be caused by perceptions about PWID among providers: low rates of adherence to the disease evaluation process and medications, ongoing substance abuse, and risk for reinfection via syringe sharing, leading to a delay in progressing through the HCV care cascade ultimately because of concerns about treating HCV among PWID. Only 20% of a sample of HCV specialists in Canada indicated they would consider treating active PWID for HCV (14). Similarly, among US physicians who treat substance abuse, 61% reported screening PWID for HCV, but only 9% offered treatment; after provision of training and resources, the proportion willing to treat HCV increased to 30% (15). Hesitation among physicians to treat patients with HCV affects earlier steps in the care cascade, including diagnosis.

Various interventions have been implemented to improve the ability of health care systems to screen for HCV. One health care system implemented an intervention that prompted physicians with a reminder sticker to assess the patient for 12 HCV risk factors; 27.8% of their patient population had at least 1 risk factor, and of these patients, 55.4% were screened. Other interventions for risk-based HCV screening struggle to screen people at risk of the disease, including PWID (16).

Other barriers specific to the FQHC studied may exist. EHR-based communication between nurses and other medical team members may be inconsistent, especially compared with in-person communication. The nursing forms used in our intervention identified most confirmed PWID in need of HCV screening, but the intervention may have failed to sufficiently motivate medical providers to increase their attention to improving screening particularly within a FQHC operating with limited resources and staff. Additionally, burnout caused by EHR-related alert fatigue is well documented in the literature and is particularly prevalent among family medicine practitioners such as those providing care at an FQHC (17). In addition, patient populations served by FQHCs have socioeconomic and psychosocial barriers to adherence to medical treatment and management, including completion of laboratory tests. Additional research and resources are needed to address various obstacles to HCV screening among both patients and medical providers, including health care team communication, patient education, financial barriers, and PWID-related stigma.

New HCV screening recommendations may help to overcome some barriers related to risk-based screening (18). Shortly after this study, in March 2020, the USPSTF and CDC updated their guidelines to recommend one-time HCV screening for all adults (18,19). Additionally, these new universal screening recommendations suggest that HCV screening should take place among women during each pregnancy, and regular screening is indicated for people at high risk of HCV, such as PWID. These updates are based on the surge of HCV cases, particularly among young adults; the availability of curative therapies with greater than 90% efficacy developed in the past 6 years; and concern about the reluctance of patients to disclose stigmatizing risk factors such as IVDU (20–22). The universal screening guidelines may support more widespread implementation of HCV screening for all adults, simplify clinic processes by minimizing the need to identify risk factors before screening for HCV, reduce stigma associated with HCV screening, and perhaps even increase financial resources for screening, all of which would benefit PWID, particularly those who choose not to disclose IVDU practices.

The universal screening recommendation for regular HCV screening among PWID requires attention to IVDU as a risk factor and, thus, a one-time screening does not sufficiently meet the health care needs of PWID. Additionally, barriers such as patient and provider education and buy-in, adherence to laboratory tests ordered at a visit, and financial costs are not overcome by the universal screening recommendations.

Our study has limitations. Before implementing the intervention, education on PWID identification and documentation was provided to medical staff members. The nurses received this education before the preintervention period began. The proportion of PWID screened for HCV during preintervention may have increased because of enhanced PWID awareness among the health care team. Furthermore, it was not possible to establish a link between participation in the educational facet of the intervention and the extent to which medical providers recommended screenings to their patients. We could not control for individual variability in provider performance because of changes in staff, patient-declared primary care provider, and patients seeing different medical providers based on schedule availability. Another limitation was the use of an observational design that included all patients seen at the clinic who met the study’s inclusion criteria. The inability to randomly draw study participants from a potentially large population prevented us from ensuring that the study’s statistical tests were adequately powered. Perhaps more importantly, we drew data from a single FQHC primary care clinic. The study also could not evaluate detailed data on race and ethnicity data; the EHR reported only Hispanic or non-Hispanic ethnicity. Because we aimed to increase HCV screening among PWID presenting for medical care, some patients were seen only in 1 phase of the study (ie, preintervention or postintervention). As a result of these factors, the external validity of the study’s results cannot be established. Lastly, we counted patients who were screened for HCV before the intervention as screened after the intervention if they had a medical provider visit during postintervention. Medical providers may have identified patients who had been screened previously and decided they did not need to be screened again because of resolution of IVDU as a risk factor (ie, the patient was no longer injecting drugs) or a lack of clarity about regularly screening PWID for HCV (23). However, we cannot verify whether medical providers recommended and verified HCV screening completion.

Given the availability of curative therapies, HCV should no longer pose individual risk for chronic infection or population risk as an epidemic. However, HCV remains a major contributor to the global prevalence of disease because of low adherence to all stages of the care cascade. Although universal screening overcomes some barriers for people with a history of IVDU, PWID will require tailored interventions because of the need for regular screening in this population versus the one-time screening recommended for most adults. Furthermore, population health improvements will require reducing the volume of HCV carriers in the PWID population so that sharing syringes poses a lower risk of HCV transmission. Such a change requires HCV screening that targets populations with a high prevalence of HCV disease. Enhanced attention to PWIDs and implementing guideline-based screening is essential to provide comprehensive care to this population. Our FQHC-based HCV screening intervention increased screening modestly while elucidating variables and issues intertwined with HCV screening. Several patient- and provider-related barriers will remain despite universal screening and will require expanded dedication of time and resources. Additionally, successful progression through the HCV care cascade to ensure PWID are treated and cured is required to reduce further transmission; a public health strategy termed “treatment as prevention” (24,25).

Education and workflow changes may modestly increase the proportion of PWID screened for HCV, yet further work is needed to achieve goals established by WHO to increase screening rates by 90% by 2030 to reach the goal of HCV eradication (2). Further research must identify barriers to screening completion and address those barriers to reach this benchmark. Given the prevalence and transmission of HCV among PWID, future studies should explore both patient- and provider-related barriers to recommending and completing HCV screening in this population. Beyond screening, adherence to recommended health care follow-up and HCV treatment will require assessment. The effect of the new universal screening guidelines on screening rates remains to be seen, particularly among PWID, to reach the ultimate goal of reducing the prevalence of HCV.
